# Reviews

**Published:** 1868-04

**Authors:** 


### REVIEWS.

WE are in receipt of the following publications:

LECTURES ON DISEASES OF WOMEN—By Charles West, M. D.; Third American Edition. Published by Henry C. Lea, Philadelphia, 1867.

Mr. Lea has again presented the profession of this country with a new edition of these well known "Lectures."

Dr. West's lectures are graphic and able, and furnish an excellent and reliable guide to the practitioner in the diagnosis and treatment of "Female Diseases," and we take pleasure in recommending this third edition to our readers.

CHEMISTRY—By Wm. Thomas Brand, D. C. L., &c., and Alfred Swaine Taylor, M. D. F. R. S. Second American Edition, revised. Published by Henry C. Lea, Philadelphia, 1868.

We do not hesitate to pronounce this the ablest work on Chemistry in the English language.

The portion devoted to Organic Chemistry is full and complete, and is alone well worth the price of the book.

It is printed on good paper, with large, clear type, and we predict that it will meet with general favor from students and physicians.

ESSENTIALS OF THE PRINCIPLES AND PRACTICE OF MEDICINE—A Hand-Book for Students and Practitioners. By Henry Hartshorne, M. D. Published by Henry C. Lee, Philadelphia, 1867.

This is a very valuable little work. We trust it will be extensively used by our medical men and students.

All the latest knowledge in this branch of medicine is grouped in a clear, compact form, and it is well worth the small price at which it is furnished.

A TREATISE ON HUMAN PHYSIOLOGY—By John C. Dalton, M. D., &c. Fourth Edition, revised and enlarged. Henry C. Lea, Philadelphia, Publisher, 1867.

Dalton's Physiology is, beyond all doubt, the best work on this subject that has appeared in this country.

It is written in a clear, concise style. The chapters on the Proximate Principles, on the Blood, on Respiration, and the Nervous System, deserve special attention.

ANNUAL ABSTRACT OF THERAPEUTICS, MATERIA MEDICA, PHARMACY AND TOXICOLOGY, FOR 1867—With a Memoir on Gout, Gravel, and Urinary Calculi. By A. Bouchardat, Professor of Hygien, to the Faculty of Medicine, Paris. Translated and Edited by M. J. De Rosset, M. D., Adjunct to the Professor of Chemistry, University of Maryland. Has just been issued from the publishing house of Lindsay & Blakiston, Philadelphia.

This little work contains valuable information, and is intended to save the practitioner the annoyance of a tedious review of the subjects treated of, when professional or other engagements are pressing.

We are also in receipt, from the same house, (Lindsay & Blakiston, of Philadelphia) of a little book of 95 pages on "Plastic Surgery," by David Prince, M. D. This is a re-print from the transactions of the Illinois State Medical Society. The work is neatly executed, the subject highly interesting, and the author entitled to commendation.

